# Plasma exchange for treating anti-Yo-associated paraneoplastic cerebellar degeneration: Case report and literature review

**DOI:** 10.1097/MD.0000000000021760

**Published:** 2020-08-14

**Authors:** Feng-Qi Hu, Fu-Rong Shang, Jing-Jing Liu, Hai Yuan

**Affiliations:** a Department of Nephrology; b Department of Neurology, Xiangyang Central Hospital, Affiliated Hospital of Hubei University of Arts and Science, Xiangyang, Hubei, China.

**Keywords:** anti-Yo antibody, case report, paraneoplastic cerebellar degeneration, plasma exchange

## Abstract

**Rationale:**

Paraneoplastic cerebellar degeneration (PCD) is a rare neurodegenerative syndrome associated with antibodies targeting the Purkinje cells of the cerebellum. Most cases of anti-Yo-associated PCD occur in females, with <20 cases reported in males. Herein, we report a male patient with anti-Yo-associated PCD who was treated with plasma exchange and achieved a favorable outcome.

**Patient concerns:**

A 64-year-old man presented with progressive ataxia, gait instability, and dysuria. Electroencephalography, electromyography, brain and spinal neuroimaging, and routine laboratory examinations were all normal. The anti-neuronal antibody Anti-Yo was detected in the serum but not in the cerebrospinal fluid (CSF).

**Diagnosis:**

The patient was diagnosed with definite anti-Yo-associated PCD based on the clinical manifestations, anti-Yo was detected in the serum and response to treatment.

**Interventions:**

At beginning, the patient was treated with dexamethasone (10 mg/day for 10 days). Then, plasma exchange was performed.

**Outcomes:**

After treated with dexamethasone, no clinical improvement was noted in this patient. In the following month, his condition deteriorated. However, after two courses of plasma exchange, neurological examination showed marked improvement in gait. After four courses of plasma exchange, the patient could walk independently, the Romberg test was negative, and anti-Yo antibodies were undetectable. At the 6-month follow-up, the patients’ symptoms were relieved, and tests for anti-Yo antibodies remained negative.

**Lessons subsections:**

Treatment with plasma exchange for anti-Yo-associated male PCD patients without a concomitant tumor are recommend and need more studies.

## 1 Introduction

Paraneoplastic cerebellar degeneration (PCD) is an immune-mediated syndrome associated with antibodies targeting the Purkinje cells of the cerebellum. This syndrome usually causes severe pancerebellar dysfunction, manifesting as progressive gait ataxia, dysarthria, and nystagmus.^[1]^ Pathologically, PCD is characterized by extensive Purkinje cell loss and the presence of highly specific antineuronal antibodies in the serum and/or cerebrospinal fluid (CSF). The definitive pathogenesis of PCD remains unclear; nevertheless, a possible etiology is autoimmunity against antigens expressed in both neoplasms and normal neural tissues, which results in humoral and T cell-mediated immunoreaction in neurons.^[1]^

Up to date, more than 20 autoantibodies associated with PCD have been identified, and anti-Yo antibodies are the most common. Anti-Yo antibody, also known as Purkinje cell cytoplasmic antibody type 1 (PCA1), targets cytoplasmic antigens in the Purkinje cells of the cerebellum.^[2]^ Anti-Yo-associated PCD has been reported in cases with various tumors, such as ovarian carcinoma, cervical cancer, and breast carcinoma, and only ∼2% of all patients with anti-Yo-associated PCD are tumor-free.^[1]^ Additionally, the majority of cases of anti-Yo-associated PCD occur in females, with <20 cases reported in males to date.

Currently, there is no effective therapeutic strategy for anti-Yo-associated PCD. Some chemotherapies and immunotherapies have been attempted using corticosteroids, intravenous immunoglobulin (IVIG), plasmapheresis, and plasma exchange,^[1]^ but the clinical prognosis has remained poor. Herein, we report a case of anti-Yo-associated PCD in a male patient who was treated with plasma exchange and achieved a favorable outcome.

## 2 Case presentation

A 64-year-old man presented with a 5-day history of progressive ataxia, gait instability, and dysuria. He also complained of blurred vision, diplopia, and blepharoptosis. During these 5 days, he had fallen two times. He denied any fever, headache, syncope, or hearing loss. His previous medical history was unremarkable. On admission, neurological examination showed bilateral horizontal gaze nystagmus, cerebellar ataxic gait, and a positive Romberg test. There was no alteration in muscle strength or muscle tone. Electroencephalography and electromyography were normal; nerve conduction velocities and evoked potentials were all normal. Routine hematology and biochemistry parameters were all within normal ranges. CSF examination showed an elevated protein level (463 mg/L). Gram staining and microbiological analysis of CSF were negative. Demyelinating antibodies, antinuclear antibodies, and anti-neutrophil cytoplasmic antibodies were not detected. Tests for neostigmine, syphilis, and HIV were negative, and levels of viral hepatitis markers and tumor markers (AFP, CEA, CA 199, SCC, CA 125, PSA, and NSE) were all normal. Brain and spinal magnetic resonance imaging (MRI), as well as whole-body computed tomography (CT), showed no abnormalities.

Anti-Yo antibody IgG was detected in the serum but not in the CSF, while other antineuronal antibodies (Anti-Hu, Anti-Ri, Anti-Ma2, Anti-CV2, Anti-amphiphysin, Anti-ANNA3, Anti-Tr, Anti-PCA-2, and Anti-GAD) were not found. Esophagogastroduodenoscopy and whole-body positron emission tomography (PET) were performed and revealed no abnormalities. According to the diagnostic criteria for paraneoplastic neurological syndromes proposed by the European Federation of Neurological Societies,^[3]^ this patient was diagnosed with definite anti-Yo-associated PCD. He was treated with dexamethasone (10 mg/day for 10 days), but no clinical improvement was noted. Two weeks later, the patient presented with altered mental status, aggravated ataxia, and dysarthria. His speech was increasingly slurred, and he was unable to stand without assistance. There were also attention, concentration, and judgment deficits. Routine hematology and biochemistry parameters were within normal ranges. During the next month, he experienced a 20-lb weight loss, and his neurological deficits deteriorated. He developed anorexia, abdominal distention, drowsiness, and bilateral lateral gaze nystagmus. Plasma exchange (1.3-fold total plasma volume with fresh frozen plasma) was performed daily using the MultiFiltrate CRRT (Fresenius Medical Care, Bad Homburg, Germany) as a dialytic device. The blood flow rate was 150 mL/min, and the plasma flow rate was 20 mL/min. Low-molecular-weight heparin (5000 IU) was used for anticoagulation therapy. No adverse reaction was observed during the treatment. After two courses of plasma exchange, neurological examination showed marked improvement in gait. After four courses of plasma exchange, the patient could walk independently, the Romberg test was negative, and anti-Yo antibodies were undetectable. From the end of the fourth course of plasma exchange, oral glucocorticoid (10 mg prednisone) was administered for 3 months. After a follow-up period of 6 months, the patients’ symptoms were alleviated, the clinical examinations were normal and anti-Yo antibodies were not detected.

This study was approved by the ethics committee of Xiangyang central hospital. Written informed consent was obtained from the patient.

## 3 Discussion and conclusion

Anti-Yo-associated PCD is classified as an autoimmune disease, and the definitive pathogenic mechanism has yet to be elucidated. The most plausible hypothesis is that neoplastic cells express antigens that are also expressed in neuron cells, and the cross-reacting antibodies cause damage within neuronal cells.^[4]^ A recent study confirmed the expression of the anti-Yo antigen in the concomitant tumor.^[5]^

According to previous studies, ∼98% of patients with anti-Yo-associated PCD have a concomitant tumor.^[1]^ However, in some cases, there was no detectable underlying malignancy even on postmortem examination.^[6]^ According to the recommended diagnostic criteria for paraneoplastic neurological syndromes in 2004, independent of the presence of cancer, patients with classical neurological syndromes and detectable onconeural antibodies (anti-Yo antibodies or other antibodies) can be diagnosed with definite paraneoplastic neurological syndromes.^[3]^ A plausible explanation for paraneoplastic neurological syndromes without cancer is that the tumor has been eliminated by the immune response.^[3]^ Most cases with anti-Yo-associated PCD occur in females, and the most common coincident tumors are ovarian carcinoma, uterine cancer, and breast carcinoma. Up to date, <20 cases of anti-Yo-associated PCD have been reported in men.^[1,5]^ A recent study proposed that anti-Yo-associated PCD in male patients may be associated with adenocarcinomas of the gastrointestinal system, especially the upper gastrointestinal tract.^[5]^

We reviewed the relevant literature, and only a few cases of anti-Yo-associated PCD treated with plasmapheresis or plasma exchange have been reported (Table 1). Previous studies have provided no evidence regarding the long-term efficacy of plasma exchange for treatment of anti-Yo-associated PCD. Peterson et al reported 22 patients with anti-Yo-associated PCD who underwent at least 5 courses of plasmapheresis, and only 1 patient experienced clinical benefits.^[6]^ In another case of anti-Yo-associated PCD secondary to ovarian cancer, no clinical improvement was achieved after a total of 22 courses of plasmapheresis.^[11]^ The possible mechanism explaining the inefficacy of plasmapheresis or plasma exchange may be that the original tumor persistently produces anti-Yo antibodies and plasmapheresis/plasma exchange cannot completely eliminate these pathogens. Notably, Meloni et al reported a case of anti-Yo-associated PCD without a concomitant tumor, in which the patient's symptoms improved rapidly following treatment via plasma exchange, and the anti-Yo antibody remained undetectable during the follow-up period.^[4]^ Consistently, in the current case of anti-Yo-associated PCD, no tumor was noted, and plasma exchange led to a favorable outcome.

**Table 1:**
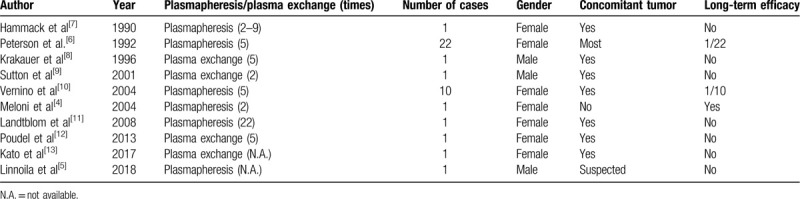
Previous cases of anti-Yo-associated PCD treated with plasmapheresis or plasma exchange.

The existing evidence shows that anti-Yo antibodies can lead to Purkinje cell death, an effect that may be prevented by eliminating the antibodies.^[14]^ Plasma exchange has been applied to treat several neuro-immunological disorders, such as Myasthenia gravis and Guillain–Barre syndrome.^[15]^ Our findings suggest that plasma exchange may be a promising approach for rapidly removing anti-Yo antibodies, and this case report highlights the efficacy of plasma exchange for managing anti-Yo-associated PCD.

We report a male case of anti-Yo-associated PCD without a concomitant tumor, which is extremely rare. More studies are needed for determining the effectiveness of plasma exchange for anti-Yo-associated male PCD patients without a concomitant tumor.

## Author contributions

FQH and FRS designed/performed most of the investigation, data analysis and wrote the manuscript; JJL contributed to acquisition of data; HY contributed to study design, analysis and interpretation of data, and critical revision of the manuscript. All the authors have read and approved the manuscript.
